# Community-based interventions to improve hepatitis C linkage to care among people with substance use disorders in Southwest Virginia: a pragmatic sequential cohort study

**DOI:** 10.1093/rescon/vmag171

**Published:** 2026-09-22

**Authors:** Mariana Gomez de la Espriella, Merly Konathapally, Sarah Henrickson Parker, Cynthia Morrow, Marrieth Rubio, Tara P Menon, Cara Ravagli, Anthony Baffoe-Bonnie

**Affiliations:** Virginia Tech Carilion School of Medicine, 2 Riverside Circle, Roanoke, VA 24016, United States; Department of Internal Medicine, Carilion Clinic, Roanoke, VA 24014, United States; Department of Internal Medicine, Mary Washington Healthcare, Fredericksburg, VA 22401, United States; Virginia Tech Carilion School of Medicine, 2 Riverside Circle, Roanoke, VA 24016, United States; Department of Internal Medicine, Carilion Clinic, Roanoke, VA 24014, United States; Fralin Biomedical Research Institute at VTC, Roanoke, VA 24016, United States; Virginia Tech Carilion School of Medicine, 2 Riverside Circle, Roanoke, VA 24016, United States; Virginia Department of Health, Richmond, VA 23219, United States; Department of Internal Medicine, Carilion Clinic, Roanoke, VA 24014, United States; Virginia Tech Carilion School of Medicine, 2 Riverside Circle, Roanoke, VA 24016, United States; Department of Internal Medicine, Carilion Clinic, Roanoke, VA 24014, United States; Virginia Tech Carilion School of Medicine, 2 Riverside Circle, Roanoke, VA 24016, United States; Department of Internal Medicine, Carilion Clinic, Roanoke, VA 24014, United States; Department of Internal Medicine, Central Virginia VA Health Care System, Richmond, VA 23249, United States

**Keywords:** hepatitis C, substance use disorders, linkage to care, telehealth, community-based interventions

## Abstract

In 2024, the reported hepatitis C virus (HCV) case rate in Roanoke City was nearly three times the rate of the state of Virginia, reflecting the disproportionate impact of the opioid epidemic in the region. Despite highly effective direct-acting antiviral therapies, substantial barriers to linkage to care (LTC) persist among individuals with substance use disorders (SUDs). While prior studies suggest that patient navigation and financial support may improve LTC, evidence from rural and underserved settings remains limited. This study aims to evaluate community-based interventions designed to improve HCV LTC among individuals with SUD in Southwest Virginia. We conducted a pragmatic, non-randomized, sequential cohort pilot study to evaluate the impact of iteratively refined community-based interventions on LTC. A total of 60 adults with detectable HCV RNA and SUD were enrolled sequentially into four cohorts (control and three intervention groups). All participants received peer support; intervention components were modified across cohorts to address observed barriers to care. The primary outcome was attendance at an outpatient HCV visit within 180 days of enrolment. LTC was 3/15 (20%) in both the control group and Intervention 1, increasing to 6/15 (40%) in Intervention 2 and 11/15 (73%) in Intervention 3. Exploratory analyses demonstrated significantly higher attendance in Intervention 3 than in the control group (73% vs. 20%, *P* = .009). Telehealth utilization and provision of prepaid phones were incorporated in the final cohort, which demonstrated the highest LTC rate. Multicomponent community-based interventions were associated with improved LTC among individuals with SUD. Although the non-randomized sequential design limits causal attribution, the progressive improvement across cohorts provides preliminary support for a potential benefit. Interventions incorporating community navigation, telehealth access, and communication support may help address barriers to HCV treatment engagement in underserved communities.

## Introduction

Approximately 2.5 million US adults were estimated to have current hepatitis C virus (HCV) infection during 2017–2020, with a separate model adjusting for the underrepresentation of people who inject drugs placing the number at approximately 4 million current infections during the same period [1]. In 2024, the reported HCV case rate was 47.7 per 100 000 adults in Virginia. In Roanoke City, Southwest Virginia, the case rate was more than double the state rate at 124.6 per 100 000 adults [2]. Injection drug use is the greatest risk factor for HCV infection and remains the most common method of HCV transmission in the United States [3]. Fortunately, direct-acting antiviral (DAA) treatment for HCV is a curative 8–12-week course of daily oral medication and is recommended for nearly all patients with HCV infection, except those with a life expectancy of less than 1 year that cannot be remediated by HCV treatment or other directed therapy [4, 5].

Despite this curative treatment, significant barriers remain in linkage to care (LTC) and treatment uptake. National data indicate that treatment rates remain substantially below HCV elimination targets, with only about one in three individuals receiving treatment, and this is even lower among people with Medicaid [6]. Although Virginia Medicaid has removed many restrictions to treatment, including prior authorizations and substance use requirements, treatment initiation remains low and demonstrates the need for additional intervention [7–9]. These barriers may be amplified in rural and underserved communities, where the effects of the opioid epidemic intersect with transportation limitations, socioeconomic disparities and limited access to specialist care. Together, these factors may further impede treatment engagement [10].

Experts recommend continued evaluation of novel models of care, such as peer-based services, and identification of barriers and facilitators by patients and providers [11]. Access alone is insufficient; interventions must be tailored to local barriers and care context. Completion of the first medical visit following diagnosis is a significant step in the HCV care continuum and represents an area for targeted improvement.

Several interventions have been explored to improve LTC, including care coordination, patient navigation, integrated care models, peer support, and telehealth-based approaches. Systematic review data suggest that these multicomponent interventions can improve referral, attendance, and treatment initiation following HCV diagnosis [12]. Programs that include a peer specialist or patient navigator have shown improvements in LTC following an HCV diagnosis in larger cities such as Philadelphia and Baltimore [13, 14]. Incentives have improved care engagement in primary care clinics, outpatient specialist settings, and community-based programs [12, 15]. Telehealth has simplified the HCV care continuum without being inferior to in-person visits and may improve engagement [16]. These studies suggest that multicomponent interventions may improve treatment linkage while highlighting persistent barriers to HCV care engagement.

However, most evidence is derived from urban or academic settings, and evidence evaluating these strategies in rural and underserved communities remains limited. Rural implementation may require additional adaptations to address long travel distances, limited specialist availability, inconsistent access to communication, and limited availability of healthcare services. To address this gap, we conducted a pragmatic, sequential cohort study evaluating community-based interventions designed to improve LTC among individuals with substance use disorder (SUD) in Southwest Virginia. We hypothesized that multicomponent interventions incorporating peer support, incentives, and flexible care delivery models would improve linkage to HCV care in this population.

## Methods

### Study design

We conducted a pragmatic, non-randomized, sequential cohort study evaluating community-based interventions designed to improve linkage to HCV care among individuals with SUD. Interventions were modified sequentially across four cohorts in response to observed linkage-to-care outcomes and operational barriers identified in the preceding cohort; the Control cohort served as the baseline comparison group. Cohort size was selected pragmatically, based on feasibility and staffing capacity, and to allow timely review of outcomes and refinement between cohorts.

The study included 60 participants enrolled sequentially into four cohorts of 15 participants each: Control (12 July 2022–10 October 2022), Intervention 1 (25 October 2022–25 January 2023), Intervention 2 (25 April 2023–15 October 2024), and Intervention 3 (6 November 2024–29 January 2025). Enrolment transitioned to the subsequent cohort once 15 participants had been enrolled in the preceding cohort; the interval between cohorts was 15 days (Control to Intervention 1), 90 days (Intervention 1 to Intervention 2), and 22 days (Intervention 2 to Intervention 3). The prolonged enrolment period for Intervention 2 reflected slower recruitment and staffing limitations.

### Setting and participants

Eligible participants were adults aged 18 years or older with a positive HCV antibody test with detectable HCV RNA, a history of SUD, and active illicit drug use at the time of enrolment. Exclusion criteria included prior treatment of HCV infection and pregnancy. Treatment-experienced individuals were excluded because the study was designed to evaluate a simplified treatment pathway for treatment-naive patients; retreatment may require additional evaluation and individualized regimen selection, which were beyond the scope of this pilot study.

Participants were recruited from inpatient settings within a tertiary hospital system located in Southwest Virginia and through a local harm-reduction organization that provides sterile syringes, naloxone, HCV point-of-care testing, linkage to substance use treatment, and social services. In the inpatient setting, potentially eligible participants were identified through an electronic health record (EHR) report. In the outpatient setting, participants were identified by peer recovery specialists (PRS) through HCV testing performed at the local harm reduction organization. Eligible participants in both settings were approached by a member of the research team, informed about the study, and consented before enrolment and referral for HCV care. All participants provided informed consent, with institutional review board approval.

Peer Recovery Specialists (PRS) were non-clinical peer support staff with experience of substance use and recovery. They assisted with participant intake, assessment of social needs, coordination of confirmatory laboratory testing, appointment coordination, transportation support, and facilitation of outpatient visits. In the inpatient setting, a team of three PRS remained involved throughout the study period, with each participant assigned to one PRS. Beginning with Intervention 2, two additional PRS from a local ­outpatient harm-reduction organization were added to support outpatient recruitment and LTC. The same one-PRS-per-participant assignment model was used in the ­outpatient setting.

Initial HCV treatment visits were conducted by an advanced practice provider (APP) or a physician with expertise in infectious diseases and hepatitis C management, in accordance with institutional standard-of-care practices. Participants in Intervention 3 could choose between an in-person or telehealth visit.

### Interventions

The interventions were cumulative across cohorts and reflected progressive modifications to the care pathway over time. Intervention components were introduced sequentially in response to barriers identified during program implementation, including transportation limitations, inconsistent phone access, and difficulty attending in-person appointments. Key Intervention components included contingency vouchers, community-based peer navigation provided by peer-recovery specialists, telehealth visits, and prepaid mobile phones.

Each voucher was valued at $25 and was provided upon completion of specified study or treatment milestones, as detailed in Fig. 1. The timing and frequency of voucher distribution were modified across intervention cohorts based on attendance observed in the preceding cohort.

**Figure 1:**
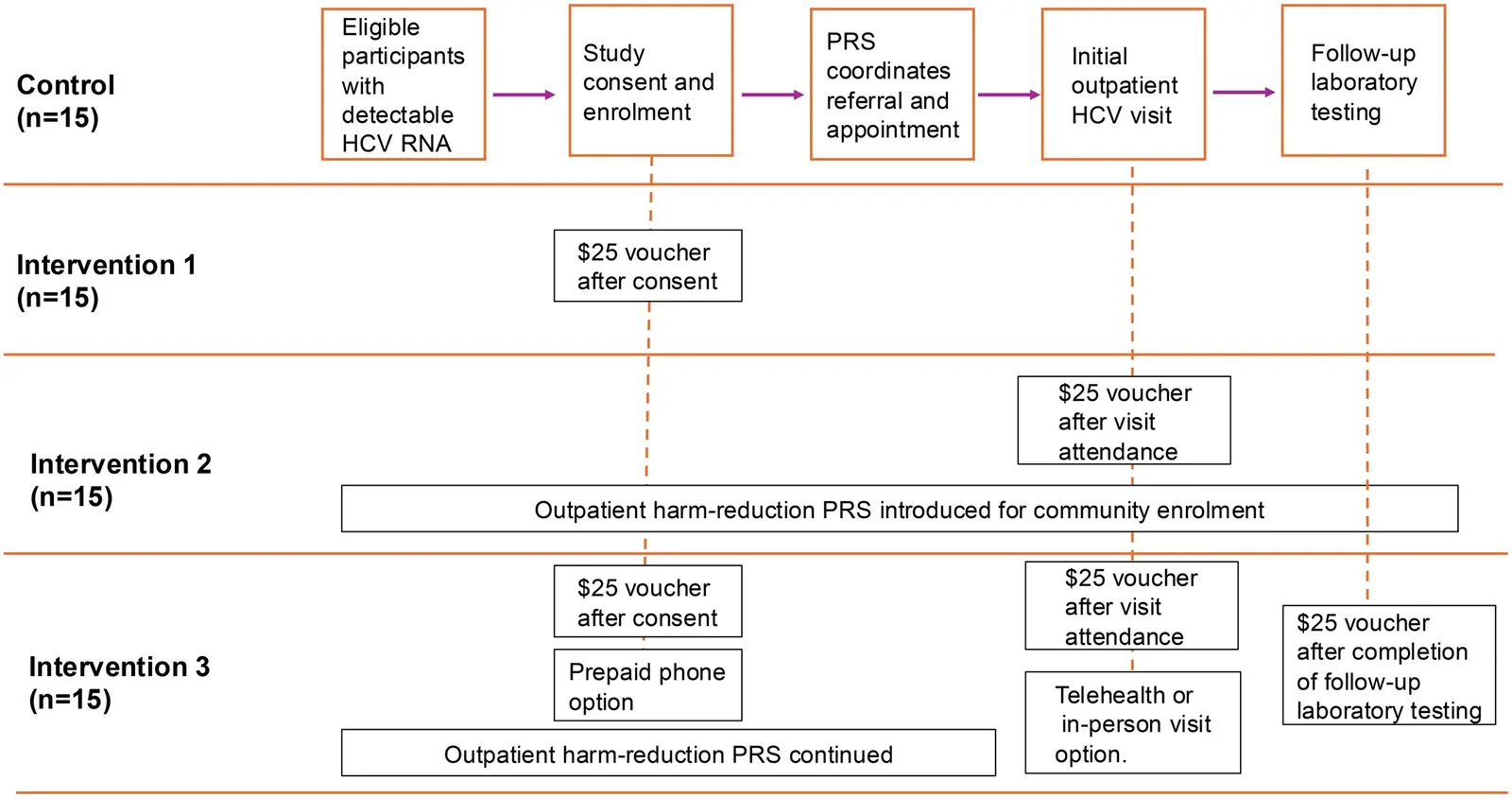
Sequential intervention components and milestone-based voucher distribution across study cohorts. Intervention components across four sequential cohorts. All participants received Peer Recovery Specialist support. Intervention 1 added a $25 voucher at consent; Intervention 2 provided a $25 voucher after the initial HCV visit and added outpatient harm-reduction Peer Recovery Specialists; Intervention 3 provided $25 vouchers at consent, the initial HCV visit, and follow-up laboratory testing, with prepaid phones when needed and participant choice of telehealth or in-person care. HCV: hepatitis C virus; PRS: Peer Recovery Specialist.

All participants received standard support from a peer recovery specialist (PRS), including care coordination, linkage to services, and referral to the infectious disease clinic.

Participants in the *Control cohort* received PRS support alone, did not receive vouchers, and were referred for standard-of-care outpatient HCV treatment.

In *Intervention 1*, participants received contingency vouchers in addition to PRS support. These vouchers served as modest financial incentives intended to reduce barriers to care engagement and encourage attendance at scheduled appointments and treatment-related activities.

In *Intervention 2*, participants received contingency vouchers and PRS support, with the addition of an outpatient peer recovery specialist from a local harm reduction organization. The timing and frequency of voucher distribution are detailed in Fig. 1. Each participant worked with one PRS based on enrolment setting: an inpatient PRS for participants enrolled through the hospital, and an outpatient PRS for those enrolled through the harm reduction organization.

In *Intervention 3*, participants received all prior intervention components, along with the option to receive a prepaid mobile phone and a choice between telehealth and in-person visits. Prepaid phones were provided to participants with limited or inconsistent access to communication, facilitating contact with healthcare teams and appointment coordination. Telehealth visits were incorporated to improve access to follow-up care for participants facing transportation, scheduling, or geographic barriers to attending in-person visits. Participants chose whether to complete their HCV visit by telehealth or in person.

### Outcome measures

The primary outcome was linkage to HCV care, defined as attendance at an outpatient HCV-related clinical visit within 180 days of enrolment. Non-attendance was categorized as no-show, left AMA (departure against medical advice before referral was completed), cancelled, out of state (relocation outside the catchment area), loss of Medicaid eligibility, or death before LTC.

EHR review was conducted for an additional 11 months after enrolment closed to capture DAA prescriptions and HCV RNA testing occurring through either the study-defined pathway or external care encounters.

Secondary outcomes were DAA prescribing and virologic response. DAA prescribing was classified as within the study pathway (LTC followed by prescription at the study-affiliated healthcare system) or outside the pathway (see Table S1). Virologic response was assessed using HCV RNA testing at least 12 weeks and within 6 months after DAA prescription; undetectable HCV RNA during this interval was classified as documented virologic response.

### Statistical analysis

No formal power calculation was performed given the pilot nature of this study; cohort size (*n* = 15) was determined by feasibility. Continuous variables were compared across cohorts using the Kruskal-Wallis test; categorical variables were compared using Fisher’s exact test. LTC in each intervention cohort was compared with Control using Fisher’s exact test, with risk differences, relative risks, and 95% confidence intervals (Wald method). A Cochran–Armitage test assessed trend in LTC across the four ordered, cumulative cohorts. Sensitivity analysis excluded participants who died or relocated out of state before the 180-day observation window closed. All tests were two-sided (alpha = 0.05). Missing data were not imputed; denominators are reported with all percentages. Analyses used Stata (version 19.5; Stata Corp LLC, College Station, TX).

## Results

### Participant characteristics

Baseline characteristics are summarized in Table 1. Median age was 38 years (IQR 34–49; range 23–65) overall. Age differed significantly across cohorts (Kruskal-Wallis, *P* = .025), with Intervention 3 having the highest median age (47 years vs. 36–37 years in earlier cohorts). Sex (40–73% male across cohorts; Fisher’s exact *P* = .35) and race (92% White overall; *P* = .84) did not differ significantly. Participants were recruited through study sites in Southwest Virginia, with most residing in Roanoke City, Roanoke County, and surrounding rural communities; a small number resided elsewhere in Virginia and neighbouring states.

**Table 1:** Demographic characteristics of control, intervention 1, intervention 2, and intervention 3 participants. This table compares baseline demographic characteristics across the control and three sequential intervention cohorts, each containing 15 participants, and for the overall sample of 60 participants.

Characteristic	Control (*n* = 15)	Intervention 1 (*n* = 15)	Intervention 2 (*n* = 15)	Intervention 3 (*n* = 15)	Overall (*n* = 60)	***P*-value** ^a^
Age (years)						
Median (IQR)	37 (32–45)	36 (32–42)	36 (30–44)	47 (40–60)	38 (34–49)	.025
Sex, *n* (%)						
Male	6 (40)	9 (60)	8 (53)	11 (73)	34 (57)	.35
Female	9 (60)	6 (40)	7 (47)	4 (27)	26 (43)	
Race, *n* (%)						
White	15 (100)	14 (93)	13 (87)	13 (87)	55 (92)	.84
Black	0 (0)	0 (0)	1 (7)	1 (7)	2 (3)	
More than one race	0 (0)	1 (7)	1 (7)	1 (7)	3 (5)	
County of residence, *n* (%)^b^						
Roanoke City	5 (33)	10 (67)	8 (53)	5 (33)	28 (47)	–
Roanoke County	4 (27)	0 (0)	0 (0)	1 (7)	5 (8)	–
Other^c^	6 (40)	5 (33)	7 (47)	9 (60)	27 (45)	–

Data are presented as median (interquartile range [IQR]) or *n* (%).

a Kruskal–Wallis test for age; Fisher’s exact test (*r* × *c*) for sex and race.

b County comparison not formally tested given geographic rather than baseline-risk relevance.

c Includes Alexandria City, Bedford County, Botetourt County, Franklin County, Greenbrier County, Giles County, Henry County, McDowell County, Mercer County, Montgomery County, Pocahontas County, Richmond City, Salem City, Tazewell County, and Washington County.

### Primary outcome: linkage to care

LTC increased progressively across cohorts: 3/15 (20.0%) Control; 3/15 (20.0%) Intervention 1; 6/15 (40.0%) Intervention 2; 11/15 (73.3%) Intervention 3 (Fig. 2). Relative to Control, linkage was unchanged in Intervention 1 (RD 0.0%, 95% CI −28.6 to 28.6%; RR 1.00, 95% CI 0.24–4.18) and Intervention 2 (RD 20.0%, 95% CI −12.0 to 52.0%; RR 2.00, 95% CI 0.61–6.55), but significantly higher in Intervention 3 (RD 53.3%, 95% CI 23.2–83.5%; RR 3.67, 95% CI 1.27–10.55; *P* = .009). A Cochran-Armitage trend test indicated an increasing trend across cohorts (Z = 3.21, *P* = .001; Table 2).

**Figure 2:**
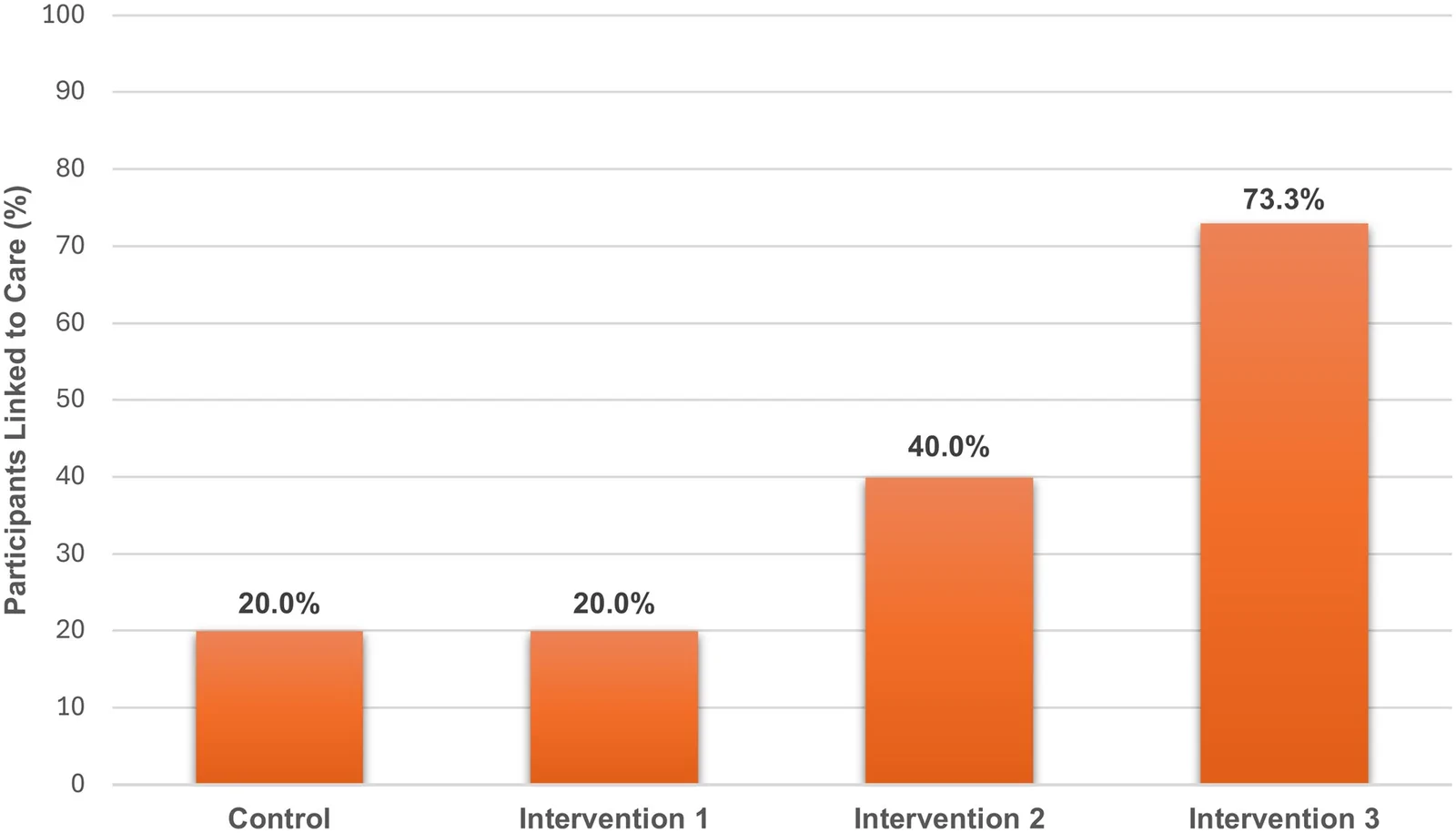
Linkage to HCV care across the four sequential study cohorts. LTC was defined as attendance at an outpatient HCV-related clinical visit within 180 days of enrollment. Rates increased from 20.0% in the Control cohort to 73.3% in Intervention 3. HCV: hepatitis C virus.

**Table 2:** LTC across cohorts. This table presents linkage-to-care outcomes across the control and three sequential intervention cohorts. The primary analysis includes all enrolled participants and reports risk differences and relative risks using the control cohort as the reference. The sensitivity analysis excludes participants who died or relocated out of state before linkage could be assessed.

Cohort	Primary analysis *n/N* (%)	Risk Difference % (95% CI)^a^	Relative risk (95% CI)^a^	***P*-value** ^b^	**Sensitivity analysis *n/N* (%)** ^c^
Control	3/15 (20.0)	Reference	Reference	—	3/11 (27.3)
Intervention 1	3/15 (20.0)	0.0 (−28.6 to 28.6)	1.00 (0.24–4.18)	1.000	3/14 (21.4)
Intervention 2	6/15 (40.0)	20.0 (−12.0 to 52.0)	2.00 (0.61–6.55)	0.427	6/15 (40.0)
Intervention 3	11/15 (73.3)	53.3 (23.2–83.5)	3.67 (1.27–10.55)	0.009	11/14 (78.6)

Data are presented as *n/N* (%).

CI: confidence interval; HCV: hepatitis C virus; RD: risk difference; RR: relative risk.

a Effect estimates use the Control cohort as the reference; risk differences are expressed as percentage-point differences.

b *P*-values were calculated using Fisher’s exact test. The Cochran–Armitage test indicated an increasing trend across the ordered cohorts (*Z* = 3.21, *P* = .001).

c The sensitivity analysis excluded participants who died or relocated out of state before linkage could be assessed. Intervention 3 remained significantly different from Control (*P* = .017).

Restricting the denominator to participants evaluable for the outcome (excluding those who died or relocated out of state) yielded similar rates: 3/11 (27.3%) Control, 3/14 (21.4%) Intervention 1, 6/15 (40.0%) Intervention 2, 11/14 (78.6%) Intervention 3, with the Intervention 3 vs. Control difference remaining statistically significant (*P* = .017; Table 2).

Within Intervention 3, 11 participants attended an HCV visit, including eight via telehealth and three in person. Eight of the nine participants scheduled for telehealth completed the visit. In addition, 8/10 (80%) participants who selected a prepaid phone attended their visit versus 3/5 (60%) who did not (RD 20.0%; *P* = .56).

Among the participants who were not linked to care, the documented reasons were: no-show (*n* = 28), death before visit (*n* = 3), relocation out of state (*n* = 3), departure against medical advice before referral completion (*n* = 2), and cancellation (*n* = 1).

### Secondary outcomes: DAA prescribing and virologic response

Overall, DAA therapy was prescribed to 29 of 60 participants (48.3%). All 10 prescriptions in Intervention 3 were issued within the study pathway (10 of 10). The corresponding proportions were lower in the earlier cohorts: 3 of 6 in the Control group, 3 of 6 in Intervention 1, 2 of 7 in Intervention 2; the remainder were prescribed outside the study pathway (Supplementary Table 1).

Among participants prescribed DAA therapy, documented follow-up HCV RNA testing ranged from 2/7 (28.6 %) in Intervention 2 to 5/6 (83.3%) in the Control cohort. Overall, 10/12 participants with follow-up testing had documented virologic response (Table 3).

**Table 3:** DAA prescribing and documented virologic response by study cohort. This table summarizes DAA treatment prescription and documented virologic outcomes across the four study cohorts. It presents the number and proportion of enrolled participants prescribed DAA therapy, participants whose prescriptions were issued within the study pathway, treated participants with documented HCV RNA assessment, and assessed participants who achieved undetectable HCV RNA (virologic response).

Cohort	DAA prescribed *n/N* (%)	Prescribed within the study pathway *n*/*N* (%)	HCV RNA assessed *n*/*N* (%)	Virologic response achieved *n*/*N* (%)
Control	6/15 (40.0)	3/6 (50.0)	5/6 (83.3)	4/5 (80.0)
Intervention 1	6/15 (40.0)	3/6 (50.0)	2/6 (33.3)	1/2 (50.0)
Intervention 2	7/15 (46.7)	2/7 (28.6)	2/7 (28.6)	2/2 (100.0)
Intervention 3	10/15 (66.7)	10/10 (100.0)	3/10 (30.0)	3/3 (100.0)
Overall	29/60 (48.3)	18/29 (62.1)	12/29 (41.4)	10/12 (83.3)

Data are presented as *n*/*N* (%), with the relevant denominator varying by outcome.

DAA: direct-acting antiviral; HCV: hepatitis C virus.

## Discussion

This pragmatic sequential cohort study reflects the challenges of treating HCV infection among individuals with SUD in real-world settings, where care pathways often require continuous adaptation to address evolving barriers to engagement. LTC improved across intervention phases, with the largest gains observed after the addition of community-based navigation, telehealth, and communication support. Rather than evaluating a fixed intervention package, this study assessed an adaptive implementation process in which barriers identified during one phase informed modifications in ­subsequent phases. This approach may be particularly relevant in rural and underserved settings, where barriers to HCV care are ­heterogeneous and may change over time.

Voucher-based incentive approaches, including contingency management models, have demonstrated the potential to improve HCV linkage-to-care outcomes, particularly attendance at initial treatment appointments [15, 17]. A systematic review found some evidence of improvements in testing and LTC, although the quality and consistency of evidence vary [18]. In our study, the introduction of vouchers in Intervention 1 was not associated with improved LTC, compared with the Control cohort. This finding suggests that modest financial incentives alone may be insufficient to overcome the structural, behavioural, and psychosocial barriers affecting HCV care engagement among individuals with SUD.

The improvement in LTC was primarily driven by the Intervention 3 cohort, with a stepwise increase observed across sequential intervention phases. The sensitivity analysis excluding participants who died or relocated out of state yielded a similar pattern of LTC across cohorts. These findings are consistent with prior studies demonstrating improved HCV treatment engagement through patient navigation, incentive-based strategies, telehealth, and multicomponent interventions in vulnerable populations [12, 13, 17, 19]. However, unlike studies evaluating fixed-linkage interventions, our intervention model evolved in response to barriers identified during clinical implementation. The progressive improvements observed across cohorts suggest that addressing multiple barriers simultaneously may be more effective than interventions targeting a single aspect of care engagement. Similar patterns have been reported in HIV care models, where bundled linkage interventions and peer-delivered case management significantly improved treatment initiation and long-term retention in care [20]. Collectively, these findings suggest that proactive, personalized linkage strategies designed to reduce barriers to care may facilitate downstream treatment engagement.

Linkage was numerically higher in Intervention 2 after the addition of community-based PRS support, although the difference from Control was not statistically significant. The largest improvement was observed in Intervention 3, when telehealth and prepaid phones were added to the prior intervention components.

Telehealth was one component of the final intervention phase. Eight of nine scheduled telehealth visits in Intervention 3 were completed, suggesting that reducing transportation and scheduling barriers may have contributed to improved linkage-to-care outcomes. Prior studies have shown that telehealth-based HCV care integrated into opioid treatment or peer-navigation programs can improve treatment engagement among underserved and rural populations [16, 21]. In this study, telehealth may have increased flexibility and reduced barriers to initial engagement in care.

Communication barriers, including inconsistent phone access, represented a major obstacle to continuity of care. Similar patterns have been reported in studies evaluating prepaid phone distribution and mobile health interventions among individuals with SUD, where improved communication access was associated with greater healthcare engagement and telemedicine utilization [22]. The provision of prepaid phones in Intervention 3 was intended to improve communication among participants, peer recovery specialists, and clinical teams to coordinate appointments and troubleshoot barriers. Although the independent effect of phone access cannot be determined, enhanced communication may have contributed to the higher linkage-to-care rates observed in the final cohort.

DAA prescribing increased in parallel with LTC, supporting an association between initial care engagement and subsequent access to treatment. Within this study-defined care pathway, DAA prescriptions increased from 20% in the Control cohort and Intervention 1 to 67% in Intervention 3. During the 11-month follow-up period, chart review identified additional DAA prescriptions outside the study-defined pathway in earlier cohorts, but none in Intervention 3. These findings suggest that participants in earlier cohorts may have relied more heavily on fragmented or external care pathways, whereas the final intervention cohort achieved greater consolidation of treatment within the structured care pathway.

Traditional cascade metrics may underestimate treatment engagement and viral clearance in real-world settings, particularly among individuals with SUD who experience fragmented healthcare access and inconsistent follow-up [23]. In this study, nearly half of all participants ultimately received DAA prescriptions through either the study-defined pathway or external care. This finding highlights the dynamic nature of HCV treatment engagement and suggests that linkage and treatment outcomes may not be fully captured when analyses are limited to formal specialty care pathways.

### Strengths and limitations

This study has several strengths. Its pragmatic design evaluated linkage interventions under real-world conditions among individuals with SUDs in rural and underserved communities. The sequential adaptive approach allowed intervention components to be refined in response to barriers identified during implementation. Collaboration among hospital-based services, community harm reduction organizations, and Peer Recovery Specialists reflected the complexity of delivering HCV care. Reporting effect estimates with confidence intervals, conducting a sensitivity analysis, and extending EHR review beyond the enrolment period strengthened the transparency and completeness of outcome assessment. Prepaid phones and financial incentives were supported through grant funding, demonstrating the feasibility of incorporating these strategies into a community-based HCV linkage-to-care program. Although a formal cost-effectiveness analysis was beyond the scope of this study, these interventions addressed common barriers to care, including unreliable communication and transportation.

This study also has several limitations. The small sample size, absence of a concurrent control group, sequential non-randomized design, and cumulative addition of intervention components limit definitive causal attribution and prevent determination of the independent effects of individual components. Temporal confounding may have occurred because cohorts were enrolled over approximately 2.5 years, during which enrolment duration, clinical practices, program infrastructure, and available follow-up time varied. Baseline demographic differences across cohorts may also have introduced residual confounding and influenced linkage-to-care outcomes. Follow-up HCV RNA testing was incomplete, particularly in Intervention 3, potentially reflecting loss to follow-up and shorter accumulated follow-up time. Because treatment completion dates were not consistently available, the assessment window was defined relative to DAA prescription, rather than treatment completion. Consequently, these findings represent documented virologic response and should not be interpreted as formal SVR-12. Recruitment through a single regional health system and community harm-reduction organization may also have introduced selection bias and limited generalizability to other populations and care settings. Furthermore, broader implementation of the interventions will require sustainable funding and adaptation to local barriers, resources, and policy contexts.

Despite these limitations, iterative, multicomponent modifications to the HCV care pathway were associated with substantial improvements in LTC among individuals with SUDs. The findings suggest that an adaptable approach, informed by locally identified barriers and available resources, may improve HCV treatment engagement in underserved communities. In this study, the approach included community-based navigation, telehealth access, reliable communication support, and peer-based care coordination. Larger studies are needed to evaluate the scalability, long-term sustainability, and cost-effectiveness of these strategies; determine the contributions of individual components and assess their effects on treatment initiation, completion, and cure.

## Conclusions

In this pragmatic sequential cohort study, LTC improved substantially across intervention phases, with the highest rates observed in the final cohort. Iterative modifications to the care pathway, including community-based navigation, telehealth access, and improved communication support, were associated with greater engagement in HCV care among individuals with SUD. These findings suggest that adaptive, multi-component interventions may help address barriers to HCV treatment in underserved communities. Larger studies are needed to identify the most effective and scalable strategies for improving treatment engagement, completion, and cure outcomes.

## Supplementary Material

vmag171_Supplementary_Data

## Data Availability

The data underlying this article cannot be shared publicly because they contain potentially identifiable health information from a small study population and are subject to institutional review board (IRB) and privacy restrictions. De-identified data that support the findings of this study may be made available by the corresponding author upon reasonable request, subject to Institutional Review Board approval and applicable data sharing agreements.

## Clinical trial registration

Registered at ClinicalTrials.gov: NCT05397067.
