# Supplementary material for: Community-based interventions to improve hepatitis C linkage to care among people with substance use disorders in Southwest Virginia: a pragmatic sequential cohort study
Source: Res Connect. 2026 Sep 22;1(4):vmag171. doi: 10.1093/rescon/vmag171 (PMC13657273; doi:10.1093/rescon/vmag171)
Supplement: Supplementary file 1 [file vmag171_supplementary_data.docx]

**Supplementary Table S1. DAA prescribing by study cohort and care pathway.** Data are presented as n/N (%). The denominator for DAA therapy is all enrolled participants. The denominator for the prescribing pathway is the number of participants prescribed DAA therapy. Prescribing within the study pathway was defined as linkage to HCV care followed by DAA prescription within the study-affiliated healthcare system.

| Cohort | Prescribed DAA n/N (%) | Prescribed within study pathway, n/N (%) | Prescribed outside study pathway, n/N (%) |
| --- | --- | --- | --- |
| Control | 6/15 (40.0) | 3/6 (50.0) | 3/6 (50.0) |
| Intervention 1 | 6/15 (40.0) | 3/6 (50.0) | 3/6 (50.0) |
| Intervention 2 | 7/15 (46.7) | 2/7 (28.6) | 5/7 (71.4) |
| Intervention 3 | 10/15 (66.7) | 10/10 (100.0) | 0/10 (0.0) |
| Overall | **29/60 (48.3)** | **18/29 (62.1)** | **11/29 (37.9)** |

*Abbreviation: DAA, direct-acting antiviral.*
